# Epidemiological profile and obstetric outcomes of patients with peripartum congestive heart failure in Taiwan: a retrospective nationwide study

**DOI:** 10.1186/s12884-017-1486-2

**Published:** 2017-09-12

**Authors:** Ying-Jen Chang, Chung-Han Ho, Jen-Yin Chen, Ming-Ping Wu, Chia-Hung Yu, Jhi-Joung Wang, Chia-Ming Chen, Chin-Chen Chu

**Affiliations:** 1 Department of Anesthesiology, Chi Mei Medical Center, 901 Zhonghua Road, Yongkang District, Tainan City, 710 Taiwan; 2 Department of Medical Research, Chi Mei Medical Center, 901 Zhonghua Road, Yongkang District, Tainan City, 710 Taiwan; 3 Department of Pharmacy, Chia Nan University of Pharmacy and Science, Tainan, Taiwan; 4 Department of Senior Citizen Service Management, Chia Nan University of Pharmacy and Science, Tainan, Taiwan; 5 Division of Urogynecology and Pelvic Floor Reconstruction, Department of Obstetrics and Gynecology, Chi Mei Medical Center, 901 Zhonghua Road, Yongkang District, Tainan City, 710 Taiwan; 6 Center of General Education, Chia Nan University of Pharmacy and Science, Tainan, Taiwan; 7 Department of Recreation and Health-Care Management, Chia Nan University of Pharmacy and Science, Tainan, Taiwan; 8 Division of Women’s and Children’s Anesthesia, Department of Anesthesiology, Chi Mei Medical Center, 901 Zhonghua Road, Yongkang District, Tainan City, 710 Taiwan

**Keywords:** Congestive heart failure, Peripartum, Epidemiological, Obstetric outcomes

## Abstract

**Background:**

During pregnancy, the hyperdynamic physiology of circulation can exacerbate many cardiovascular disorders. Congestive heart failure (CHF) usually occurs during late pregnancy, which is significantly associated with a high level of maternal and neonatal morbidities and mortalities. The profile of women who develop peripartum CHF (PCHF) is unknown. We investigated the epidemiological profiles of PCHF.

**Methods:**

In this retrospective cohort study, PCHF patients were identified using International Classification of Diseases, Ninth Revision, Clinical Modification (ICD-9-CM) codes in Taiwan’s National Health Insurance Research Database. Risk factors and obstetric outcomes were compared in women with and without PCHF.

**Results:**

From 2,115,873 birth-mothers in Taiwan between 1997 and 2013, we identified 512 with PCHF (incidence: 24.20/10^5^). More women with than without PCHF were older (≥ 35, 18.16% vs. 9.62%), and had more multifetal gestations (7.42% vs. 1.40%), gestational hypertension (HTN) (19.2% vs. 1.31%), and gestational diabetes mellitus (4.10% vs. 0.67%). After the analysis had been adjusted for confounders, the leading comorbidities associated with PCHF were structural heart diseases (adjusted odds ratio [aOR]: 67.21; 95% confidence interval [CI]: 54.29–83.22), pulmonary diseases (aOR: 13.12; 95% CI: 10.28–16.75), chronic HTN (aOR: 11.27; 95% CI: 6.94–18.28), thyroid disease (aOR: 9.53; 95% CI: 5.27–17.23), and gestational HTN (aOR: 5.16; 95% CI: 3.89–6.85). PCHF patients also had a higher rate of cesarean sections (66.41% vs. 34.46%; *p* < 0.0001).

**Conclusion:**

Maternal structural heart diseases, pulmonary diseases, thyroid disorders, and preexisting or gestational HTN are associated with a higher risk of developing PCHF. Birth-mothers with PCHF also had a higher risk of caesarean section and adverse outcomes, including maternal death. Our findings should benefit healthcare providers, and government and health insurance policy makers.

**Electronic supplementary material:**

The online version of this article (10.1186/s12884-017-1486-2) contains supplementary material, which is available to authorized users.

## Background

Congestive heart failure (CHF) is a complicated clinical syndrome that impairs ventricular pumping [1]. During pregnancy, the hyperdynamic physiology of circulation can exacerbate many cardiovascular disorders. Peripartum CHF (PCHF) usually occurs during late pregnancy or within a few months postpartum and is often significantly associated with high levels of maternal and neonatal morbidity and mortality [2, 3]. PCHF is the most common major cardiovascular complication in pregnant women with a preexisting cardiac disease [4].

PCHF can be caused by exacerbating preexisting cardiovascular [4] and systemic diseases [5–7], or by pregnancy-associated diseases [8]. Sometimes, PCHF can be idiopathic; and in this situation, it is referred to as peripartum cardiomyopathy (PPCM) [9, 10]. No matter what the etiology, PCHF is associated with adverse outcomes for birth-mothers and neonates [11]. The incidence of peripartum cardiovascular disease is growing [12], most likely because of older maternal age [13], cardiovascular risk factors (e.g., obesity, diabetes mellitus [DM], and hypertension [HTN]), and the lifespan of women with congenital heart disease [14].

There are many studies on PPCM, but studies on PCHF are still scarce. In addition, the epidemiological reports that have been published are either small-scale studies or based on small community databases [15, 16]. For such a critical disease, the existing literature is insufficient. Therefore, to help fill this gap, we used a large-scale national population-based database to investigate the long-term epidemiological profile of PCHF and determine its incidence, characteristics, and outcomes in the Taiwan population.

## Methods

### Data source

Taiwan launched a single-payer National Health Insurance (NHI) program on March 1, 1995 [17, 18]. The enrollment rate of NHI reached 99% in 1997, and has maintained at this high coverage level ever since [17]. The NHI research database (NHIRD) provides encrypted patient identification numbers, gender, date of birth, dates of admission and discharge, the ICD-9-CM (International Classification of Diseases, Ninth Revision, Clinical Modification) codes of diagnoses and surgical procedures, details of prescriptions, and costs covered and paid for by the NHI.

In Taiwan, every hospital has a coder-team to ensure the accuracy of diagnostic and management codes. Besides, Taiwan’s NHI Bureau is responsible for auditing medical payments by comprehensive review of medical records, examination reports, and results of imaging studies. If physicians fail to meet the standards for clinical practice, Taiwan’s NHI reserves the right to reject payment and can impose huge financial penalties.

We used the inpatient claims database from 1997 to 2013 because, in Taiwan, almost all deliveries occur in hospitals and almost all patients with severe diseases like heart failure are hospitalized. The dataset was released with de-identified secondary data for public research. The Taiwan National Health Research Institutes approved the present study. Moreover, because all types of personal identification were encrypted to secure patient privacy, the present study was granted an exemption from a full ethical review by the Chi Mei Medical Center Institutional Review Board (IRB: 10,505–014).

### Selection of patients and variables

**Fig. 1:**
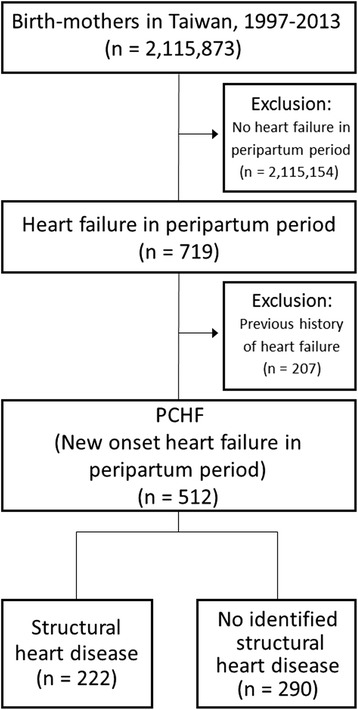
Flow chart of the creation of the study sample. Description of data: We defined peripartum congestive heart failure (PCHF) as newly developed and diagnosed CHF (ICD-9-CM code: 428.0–428.21) between 1 month before and 5 months after delivery. Women with a history of CHF or an onset of CHF beyond the peripartum period were excluded

To ensure that patients with PCHF would be comparable with patients with PPCM, we defined PCHF patients as those with newly developed and diagnosed CHF (ICD-9-CM code 428.0–428.21) between 1 month before and 5 months after delivery, based on the definition of peripartum patients in an established article on PPCM [19]. Because we focus on the new onset of PCHF, we excluded pregnant women with a history of CHF or an onset of CHF out of this defined time frame (Fig. 1).

We recorded the maternal demographic factors: age, economic status, hospital type (medical center: 1000–2500 beds/tertiary care; regional hospital: 301–999 beds/secondary care; or local hospital: < 300 beds/primary care); delivery-related factors: multifetal gestations, gestational HTN, gestational DM, and placental abnormality; comorbidities: HTN, congenital heart disease, DM, pulmonary disease, hepatic disease, renal disease, thyroid disease, anemia, malignancy, and autoimmune disease. Critical adverse cardiac events, such as ventricular tachycardia, ventricular fibrillation, and cardiac arrest, were also studied. Major comorbidities, obstetric conditions, and corresponding ICD-9-CM Codes are listed in Additional file 1.

Patients were also divided into three economic status groups based on monthly income in New Taiwan dollars (NT$): < NT$20,000; NT$20,000-NT$40,000; and > NT$40,000.

### Epidemiological profile and maternal outcomes of PCHF

We determined the incidence of PCHF between 1997 and 2013. Next, we characterized the demographics and medical conditions of the patients and estimated the associations between PCHF and possibly important related obstetrical complications: maternal death, cardiac arrest, life-threatening arrhythmia, and acute pulmonary edema.

### Statistical analysis

Significant differences in prevalence between different age groups were evaluated using a χ^2^ test. Odds ratios and 95% confidence intervals (ORs ± 95% CIs) for PCHF with and without each maternal comorbidity were determined using multivariate logistic regression adjusted for possible confounding factors: structural heart disease, pulmonary disease, liver disease, renal disease, thyroid disease, anemia, malignancy, autoimmune disease, chronic HTN, chronic DM, multifetal gestations, placental abnormality, gestational HTN, gestational DM, peripartum hemorrhage, hospital type, and economic status by monthly income. Each comorbidity was considered a single variable and was separately entered into the logistic regression model.

SAS 9.4 (SAS Institute Inc., Cary, NC, USA) was used for all data analyses. Significance was set at *p* < 0.001 (two-sided).

## Results

We identified 2,115,873 birth-mothers during 1997–2013 in Taiwan: 512 (0.0242%) patients had been diagnosed with PCHF 1 month before and within 5 months after delivery. The incidence of PCHF was 1:4133 deliveries. Birth-mothers with PCHF were significantly (*p* < 0.001) older (≥ 35 years old) than were birth-mothers without PCHF (18.16 vs. 9.62%). Additionally, they had significantly (*p* < 0.001) more comorbidities: structural heart disease (43.36% vs. 0.40%), pulmonary disease (27.93 vs. 0.34%), liver disease (2.73 vs. 0.09%), renal disease (3.71 vs. 0.03%), thyroid disease (3.32 vs. 0.10%), anemia (16.21 vs. 2.62%), malignancy (2.34 vs. 0.61%), autoimmune disease (2.93 vs. 0.06%), chronic HTN (6.64 vs. 0.05%), and DM (2.15 vs. 0.11%) (Table 1).

**Table 1:** Characteristics of parturient women with and without peripartum congestive heart failure

Characteristics	PCHF^a^	No PCHF	*P*
	*n* = 512 (%)	*n* = 2,115,361 (%)	
Age (years)			< 0.001
< 20	16 (2.79)	89,096 (4.21)	
20–34	403 (77.91)	1,822,741 (86.17)	
≥ 35	93 (18.16)	203,524 (9.62)	
Maternal comorbidities			
Structural heart disease	222 (43.36)	8463 (0.40)	< 0.001
Pulmonary disease	143 (27.93)	7152 (0.34)	< 0.001
Liver disease	14 (2.73)	1801 (0.09)	< 0.001
Renal disease	19 (3.71)	680 (0.03)	< 0.001
Thyroid disease	17 (3.32)	2064 (0.10)	< 0.001
Anemia	83 (16.21)	55,431 (2.62)	< 0.001
Malignancy	12 (2.34)	12,984 (0.61)	< 0.001
Autoimmune disease	15 (2.93)	1339 (0.06)	< 0.001
Chronic hypertension	34 (6.64)	1026 (0.05)	< 0.001
Diabetes mellitus	11 (2.15)	2316 (0.11)	< 0.001
Obstetric condition			
Multifetal pregnancies	38 (7.42)	29,697 (1.40)	< 0.001
Placenta abnormality	10 (1.95)	23,252 (1.10)	0.064
Gestational HTN	102 (19.92)	27,737 (1.31)	< 0.001
Gestational diabetes mellitus	21 (4.10)	14,101 (0.67)	< 0.001
Peripartum hemorrhage	24 (4.69)	51,363 (2.43)	< 0.001
Hospital type			< 0.001
Medical center	129 (25.20)	271,513 (12.84)	
Regional hospital	106 (20.70)	412,589 (19.50)	
Local hospital	277 (54.10)	1,431,199 (67.66)	
Monthly income (NT$)^b^			< 0.001
≤ 20,000	255 (49.80)	821,380 (38.83)	
20,000–40,000	194 (37.89)	915,011 (43.26)	
> 40,000	63 (12.30)	378,970 (17.92)	

Characteristics of birth-mothers in Taiwan with and without peripartum congestive heart failure during 1997–2013

^a^ *PCHF: * peripartum congestive heart failure

^b^ *NT$*: New Taiwan dollar

Significantly (*p* < 0.001) more birth-mothers with PCHF had multifetal gestations (7.42 vs. 1.40%), gestational DM (4.10 vs. 0.67%), and gestational HTN (19.92 vs. 1.31%), than did birth-mothers without PCHF (Table 1). However, there were no significant differences in placental abnormalities or peripartum hemorrhage between these two groups. Birth-mothers with PCHF earned significantly (*p* < 0.001) lower monthly incomes than did birth-mothers without PCHF. Moreover, significantly (*p* < 0.001) more women with PCHF sought care in medical centers (25.20 vs. 12.84%) than did women without PCHF (Table 1).

Comorbid structural heart disease raised the risk of PCHF to 190.58 times normal, and even after the analysis had been adjusted for age, hospital levels, monthly income, maternal comorbidities, and obstetric conditions, the risk was 67.21 times normal (Table 2). Comorbid pulmonary disease raised the risk of PCHF to 13.12 times normal, chronic HTN raised it to 11.27 times normal, and gestational HTN to 5.16 times normal. However, gestational DM, placental anomalies, and peripartum hemorrhage did not increase the risk (Table 2).

**Table 2:** Associations between peripartum congestive heart failure and comorbidities

Characteristics	Crude OR﻿^﻿a^ [95% CI]	Adjusted OR^b^ [95% CI]^c^
1. Maternal comorbidities		
Structural heart disease	190.58 (159.81–227.27)	67.21 (54.28–83.22)
Pulmonary disease	114.24 (94.05–138.76)	13.12 (10.28–16.75)
Liver disease	32.99 (19.36–56.23)	3.61 (1.85–7.05)
Renal disease	119.85 (75.33–190.68)	4.53 (2.28–8.99)
Thyroid disease	35.16 (21.64–57.13)	9.53 (5.27–17.23)
Anemia	7.19 (5.68–9.10)	2.99 (2.28–3.91)
Cancer	3.89 (2.19–6.89)	1.80 (0.97–3.35)
Autoimmune disease	47.65 (28.43–79.86)	2.33 (1.08–5.01)
Chronic hypertension	146.58 (102.96–208.68)	11.27 (6.94–18.28)
Diabetes mellitus	20.03 (11.01–36.46)	1.33 (0.54–3.26)
2. Obstetric conditions		
Multifetal pregnancies	5.63 (4.05–7.84)	2.13 (1.47–3.08)
Placenta anomaly	1.79 (0.96–3.35)	1.21 (0.51–2.88)
Gestational HTN	17.40 (13.92–21.75)	5.16 (3.89–6.85)
Gestational diabetes mellitus	6.37 (4.12–9.87)	1.02 (0.55–1.90)
Peripartum hemorrhage	1.98 (1.31–2.98)	0.98 (0.54–1.76)

Associations between peripartum congestive heart failure and maternal comorbidities and obstetric conditions

^a^ *OR:* odds ratio

^b^Adjusted for all confounding factors in Table 1

^c^ *CI:* confidence interval

Only 290 (0.01%) patients without a structural heart disease (reference group) vs. 222 (2.56%) with a structural heart disease developed PCHF (Table 3). Among the structural heart diseases, right-side cardiac lesions raised the risk of PCHF to 70.65 times, compared with mothers who had no structural heart disease. Left-side cardiac lesions raised it to 60.00 times, and septal wall defects raised it to 33.95 times, compared with mothers had no structural heart disease (Table 3).

**Table 3:** Incidence of peripartum congestive heart failure comorbid with Structural heart disease

Characteristics	PCHF^a^	No PCHF	Total birth-mothers	Cardiac lesion-specific PCHF frequency	Adjusted^b^ OR^c^ (95% CI)^d^
	*n* = 512	*n* = 2,115,361			
Structural heart disease					
Yes	222	8463	8685	2.56%	67.21 (54.28–83.22)
No	290	2,106,898	2,107,188	0.01%	Reference group
Location of anomaly					
Left side	23	424	447	5.15%	60.00 (35.54–101.29)
Right side	27	436	463	5.83%	70.65 (43.27–115.35)
Septal defect	112	5897	6009	1.86%	33.95 (26.56–43.38)
Others	77	2064	2141	3.60%	22.66 (16.46–31.18)
Types					
Valvular	76	1264	1340	5.67%	83.79 (61.49–114.18)
Non-valvular	290	7199	7345	1.99%	32.90 (25.88–41.82)

Incidence of peripartum congestive heart failure for birth-mothers in Taiwan comorbid with specific cardiac anomalies during 1997–2013

^a^ *PCHF*: peripartum congestive heart failure

^b^Adjusted for all confounding factors in Table 1

^c^ *O﻿R*﻿: odds ratio

^d^ *CI*: confidence interval

The risk of birth-mothers with PCHF developing major maternal adverse outcomes was high. For example, the risk ratio of acute pulmonary edema was 826.31 times for mothers with than without PCHF (Table 4). Finally, cesarean sections were more frequent, hospital stays were longer, and hospitalization costs were all significantly higher for birth-mothers with PCHF (Table 5).

**Table 4:** Major adverse outcomes of parturient women with and without peripartum congestive heart failure

Major maternal adverse outcomes	PCHF^a^	No PCHF	Relative risk (95% CI)^b^
	*n* = 512 (%)	*n* = 2,115,361 (%)	No PCHF birth-mother as reference
Acute pulmonary edema	48 (9.375)	240 (0.011)	826.31 (613.65–1112.67)
Cardiogenic shock	7 (1.367)	35 (0.002)	826.31 (368.75–1851.66)
Life threatening arrhythmia or cardiac arrest	4 (0.781)	88 (0.004)	187.80 (69.21–509.60)
Maternal death	3 (0.586)	376 (0.018)	32.96 (10.62–102.33)

Major maternal adverse outcomes of parturient women with and without peripartum congestive heart failure during 1997–2013 in Taiwan

^a^ *PCHF*: peripartum congestive heart failure

^b^ *CI*: confidence interval

**Table 5:** Cesarean sections; length of hospital stays; and hospital cost in peripartum congestive heart failure group

Variable	PCHF^a^	No PCHF	*P*
	*n* = 512	*n* = 2,115,361	
Types of delivery			< 0.001
Normal spontaneous delivery)	172 (33.59%)	1,386,467 (65.54%)	
Cesarean section	340 (66.41%)	728,894 (34.46%)	
Hospital Stay (days)			
Length of hospital stay at delivery	4.98 ± 4.38	3.51 ± 1.68	< 0.001
Total length of hospital stays, peripartum	20.15 ± 25.23	4.30 ± 8.24	< 0.001
Hospital cost [New Taiwan Dollars (NT$)]			
Hospital cost at delivery	41,474 ± 51,616	19,962 ± 9425	< 0.001
Total hospital cost, peripartum	197,386 ± 289,423	24,061 ± 33,868	< 0.001

Percentage of cesarean sections; length of hospital stays; and hospital cost in peripartum congestive heart failure group during 1997–2013 in Taiwan

^a^ *PCHF*: peripartum congestive heart failure

## Discussion

We found that delivering mothers who are ≥ 35 years old and who have preexisting comorbidities, especially structural heart disease and pulmonary disease, have a significantly higher incidence of PCHF. Moreover, women with multifetal gestations and gestational HTN were associated with a higher risk of developing PCHF than were normotensive mothers and the delivering mothers of singleton children. Additionally, delivering mothers with PCHF had a higher incidence rate of cesarean section, and they were more frequently associated with adverse outcomes—e.g., pulmonary edema, life-threatening arrhythmia, episodes of cardiac arrest, and death—than were delivering mothers without PCHF.

This is the first nationwide epidemiological study of PCHF patients. Because our data source was a 16-year population-based database, the statistical power of our analysis is stronger than that of other reports with smaller study populations.

Of the 512 patients who had been diagnosed with PCHF, 290 had not being coded any structural heart disease. Most of them were highly suspected of having PPCM. Although they were not directly coded as having PPCM (ICD-9-CM code 674.5), by definition they met the criteria for the diagnosis of PPCM [20]. We checked 2 recent PPCM cases in our hospital registration record and found they had been coded as CHF (ICD-9-CM codes 428.0–428.21), not PPCM (ICD-9-CM code, 674.5). They had been transferred to Cardiology after they had been diagnosed with heart failure. Therefore, they were coded CHF on discharge. The possible explanation is that cardiologists are more familiar with the CHF code than with the PPCM code. However, this needs to be verified.

The incidence of PPCM is estimated to be 1 in 2500 to 4000 (0.03%) total births in Canada, Europe, and the United States, 0.1% in South Africa, and 0.09% in Asia, and higher in less-developed countries like Nigeria (1%) and Haiti (0.33%) [21, 22]. However, the incidence of PCHF has never been reported. Using our large database, we first showed that the incidence of PCHF in Taiwan was 0.024% of total births, which is close to the incidence of PPCM in the United States. We could not calculate the precise incidence of PPCM in Taiwan from our present study. We could only indirectly estimate that the incidence is near or lower than 0.014%, i.e., 290 highly suspected cases of PPCM in 2,115,873 pregnant women. This incidence is much lower than that of PPCM in the United States (0.1%), but higher than it is in Japan (about 0.005%) [23].

Advanced age is always a risk for many poor gestational outcomes [24]. In our study, the percentage of PCHF patients ≥ 35 years old was twice that of women without PCHF. Being a delivering mother ≥ 35 might lead to higher incidence rates of pre-eclampsia, eclampsia, and gestational DM, which have been identified as risk factors for heart failure during pregnancy. Moreover, older women have had a substantial increase in multifetal pregnancies because of contemporary reproductive techniques [25].

Not surprisingly, delivering mothers in our study with underlying cardiac disease had a greater risk of developing PCHF, which is similar to the findings of other reports [4, 26]. After age and comorbidities had been adjusted for, women with pre-existing structural heart disease were still associated with the highest chances of developing PCHF. In our study, 2.56% of the patients with structural heart disease developed PCHF, but in the European Society of Registry of Pregnancy and Cardiac disease (ROPAC) study [4], 13.1% with a structural heart disease did. The higher incidence of heart failure in the ROPAC study might be attributable to that study’s having included heart-failure cases during the entire pregnancy rather than limiting it to peripartum period, as our study did. Our finding is closer to that of a meta-analysis [27] which reported that the frequency of heart failure was 4.8% in mothers with congenital heart disease. Except for this, the lower rate in our study might be related to differences in lifestyle, other medical conditions, or genetics [10]. Epidemiological studies [28, 29] reported that pregnant Asian women had a lower BMI, less gestational weight gain, and fewer incidences of pre-eclampsia and abnormal liver enzymes than did pregnant Caucasian women, all of which might contribute to the low incidence of both PCHF and PPCM in Asians.

We also found that 5.83% of delivering mothers with right-side cardiac lesions developed PCHF, but that 5.15% with left-side lesions and that 1.86% with a septal defect developed PCHF. This is somewhat different from the ROPAC study [4] finding that more delivering mothers with left-side lesions developed PCHF than did those with right-side lesions or a septal wall defect. In our study, after confounding factors had been adjusted for, delivering mothers with right-side lesions had a greater risk of developing PCHF (aOR: 70.65) than did those with left-side lesions or a septal wall defect, which is consistent with the findings of a prospective multicenter study in Canada [30] and a retrospective study in China [31].

Delivering mothers with valvular heart disease had a greater risk of developing PCHF than did those with cardiac lesions not involving heart valves. According to many studies [4], between 18.7 and 48.3% of pregnant women diagnosed with valvular heart disease before pregnancy develop heart failure. In our study, 5.67% of the pregnant women with valvular heart disease developed PCHF, and had an aOR 83.79 times higher (95% CI: 61.49–114.18) for developing PCHF than did delivering mothers without valvular heart disease.

Consistent with the findings of other studies [8], our findings showed that obstetric conditions like multifetal gestation, pre-eclampsia, and eclampsia contributed to the development of PCHF. Hypertension damages blood vessels already weakened by hemodynamic stress or altered by other factors associated with pregnancy [12].

Apart from cardiac anomalies, anemia and disorders of the pulmonary, renal, thyroidal, and autoimmune systems were also significantly associated with PCHF in this study. It is well documented that respiratory disturbances, fibrosis, and pulmonary hypertension is often comorbid with CHF secondary to right heart overload, which ultimately leads to heart failure [32]. Moreover, impaired renal function may cause fluid retention and increase cardiac workload. In addition, renal disease shares many risk factors—e.g., HTN and DM—with heart failure [33]. Thus, it is logical that renal disease increases the risk of PCHF. Similarly, in anemic patients, the heart must beat more vigorously and rapidly to meet the body’s demand for oxygen, which can induce cardiac de-compensation [34]. Higher levels of cytokines, in concert with higher levels of circulating autoantibodies, might attack the cardiac tissue and induce heart failure [6]. In pregnant women comorbid with thyroid problems like Graves’ disease, the high-output state and additional hemodynamic burdens might further decompensate ventricular function in the peripartum period [7].

Interestingly, we also found that pregnant women comorbid with liver disease had a risk 3.61 times greater of developing PCHF than did women not comorbid with liver disease. Those with severe liver disease might experience coagulation dysfunction, anemia, and increased cardiac effort [35]. Recently, nonalcoholic fatty liver disease has been identified as an emerging risk factor for acute heart failure [36]. This might partially explain our finding about liver disease and PCHF.

We also found that delivering mothers without PCHF had greater odds of having a cesarean section than did those without. Vaginal delivery poses a potential risk of hemodynamic fluctuations related to labor pain [13]. Women at a high risk should consider an elective cesarean section. Moreover, it is reasonable that delivering mothers with PCHF tend to seek healthcare in larger and more full-service medical centers rather than in smaller local hospitals with fewer specialist physicians.

It is worth mentioning that the total peripartum hospital stay and medical costs were substantially higher for delivering mothers with than without PCHF. We think this is clinically important because it ultimately translates into a vast amount of extra medical resources. Because medical costs are increasing worldwide, the economic aspect of health care is especially important for governments that provide government subsidized national health insurance. Therefore, our results might also benefit government and health insurance policy makers.

### Limitations

Our study has some limitations. First, because patient information in our database is de-identified, we could not use medical record reviews to confirm the ICD-9-CM diagnosis and comorbidity codes. Incorrect coding and misclassification might have biased our results. However, every hospital in Taiwan has a coder team to ensure the accuracy of diagnostic and management codes. The validity of many specific diagnoses, especially those of major diseases in inpatients in Taiwan, have been verified and reported [37, 38]. Second, every patient has at most 5 ICD-9-CM diagnostic codes for each admission; therefore, minor events and comorbid disorders might not be listed at discharge. This might underestimate the association between minor diseases and PCHF. However, major diseases, such as heart failure and chronic kidney disease, would not be ignored. Third, the medical history of patients in the NHIRD can be traced back only to January 1996. We cannot be certain whether a pregnant woman had CHF before then, and therefore cannot exclude the possibility of mistaking a patient with prior CHF as having a new case of CHF. The prevalence of some adverse outcomes might thus be overestimated. Fourth, some important sociodemographic characteristics, such as education level, nutritional status, dietary habits, alcohol drinking and tobacco use, and BMI, are not recorded in the database. Therefore, we could not adjust for these variables as contributing factors in this study. Fifth, laboratory data and image reports, e.g., echocardiograms, and some delivery records, e.g., blood loss and hemodynamic records when giving birth, are not recorded. However, major findings are expressed as ICD-9-CM codes.

## Conclusion

Maternal structural heart diseases, especially right heart and valvular heart diseases, are associated with highest risk for developing PCHF. Anemia, pulmonary, renal, and liver diseases; and preexisting and gestational HTN are also risk factors for developing PCHF. Moreover, delivering mothers with PCHF had higher ORs for adverse major maternal outcomes and cesarean sections; they also had longer hospital stays, used more medical resources, and had higher hospital costs than did delivering mothers without PCHF. Medical care providers should pay close attention to delivering mothers with high risk factors for PCHF to prevent their mortality and morbidities.

## Additional file

Additional file 1: Appendix. Major Comorbidities, Obstetric Conditions, and Corresponding International Classification of Diseases, Ninth Revision, Clinical Modification Codes. Description of data: This file shows the ICD-9 CM (International Classification of Diseases, Ninth Revision, Clinical Modification) Codes used in our study. (DOC 45 kb)

## Abbreviations

CHF: Congestive heart failure
ICD-9-CM: International Classification of Diseases, Ninth Revision, Clinical Modification
NHI: National Health Insurance
NHIRD: National Health Insurance Research Database
NT$: New Taiwan dollars
PCHF: Peripartum congestive heart failure
PPCM: Peripartum cardiomyopathy
ROPAC: Registry of Pregnancy and Cardiac disease
