# Supplementary material for: Epidemiological profile and obstetric outcomes of patients with peripartum congestive heart failure in Taiwan: a retrospective nationwide study
Source: BMC Pregnancy Childbirth. 2017 Sep 12;17:302. doi: 10.1186/s12884-017-1486-2 (PMC5596930; doi:10.1186/s12884-017-1486-2)
Supplement: Additional file 1: Appendix. — Major Comorbidities, Obstetric Conditions, and Corresponding International Classification of Diseases, Ninth Revision, Clinical Modification Codes. Description of data: This file shows the ICD-9 CM (International Classification of Diseases, Ninth Revision, Clinical Modification) Codes used in our study. (DOC 45 kb) [file 12884_2017_1486_MOESM1_ESM.doc]

Additional File 1

**Appendix.** Major Comorbidities, Obstetric Conditions, and Corresponding International Classification of Diseases, Ninth Revision, Clinical Modification Codes

| Variable | ICD-9 CM Codes |
| --- | --- |
| Maternal comorbidities |  |
| Cardiac anomaly | 394, 396, 397, 416, 424, 429, 745, 746 |
| Pulmonary disease | 415, 416, 466, 485, 486, 507, 514, 518 |
| Liver disease | 571, 573, 794 |
| Renal disease | 581, 583, 584 |
| Thyroid disease (Thyroid goiter, hyperthyroidism, | 414, 648.1 |
| hypothyroidism, etc.) |  |
| Anemia | 280, 282, 285, 648.2 |
| Malignancy | 140-239 |
| Autoimmune disease (Systemic connective disease: Systemic | 710 |
| lupus erythematosus, Sjogren’s syndrome, dermatomyositis, etc.) |  |
| Chronic hypertension | 401-405, 362.11, 437.2 |
| Diabetes mellitus | 250 |
| Obstetric Condition |  |
| Multifetal pregnancies | 651.0-651.9, V27.2-V27.7, V31-V37 |
| Placenta abnormality (Placenta previa, placenta abruption, etc) | 641.0, 641.2, 762.0-762.2 |
| Gestational diabetes mellitus | 648.0, 648.8 |
| Gestational HTN |  |
| (Pregnancy induced hypertension, pre-eclampsia, eclampsia) | 642.33, 642.4-642.7, 642.6 |
| Peripartum hemorrhage | 286, 640, 641, 666 |
| (Antepartum hemorrhage and postpartum hemorrhage) |  |
| Delivery Type |  |
| Normal spontaneous vaginal delivery | 650 |
| Cesarean section | 74.x (Procedure code) |
| Cardiac anomaly |  |
| Left side cardiac anomaly | 394-396, 746.3-746.7, 746.81 |
| Right side cardiac anomaly | 397.0, 746.0-746.2, 746.83 |
| Septal wall defect | 745 |
| Valvular heart disease | 394, 396, 397, 424 |
| Maternal Complication |  |
| Acute pulmonary edema | 428.1, 518.4 |
| Cardiogenic shock | 785.51 |
| Life threatening arrhythmia (Ventricular tachycardia and /or | 427.1, 427.4, 427.5 |
| ventricular fibrillation) or cardiac arrest |  |
| Maternal death | Death code, or dropping out of NHI after 30 days |
|  |  |

ICD-9-CM: International Classification of Diseases, Ninth Revision, Clinical Modification; NHI: Taiwan’s National Health Insurance program.
